# Integrated Transcriptomic and Proteomic Analysis Reveals Molecular Mechanisms of the Cold Stress Response during the Overwintering Period in Blueberries (*Vaccinium* spp.)

**DOI:** 10.3390/plants13141911

**Published:** 2024-07-11

**Authors:** Xin Wei, Hongguang Wang, Dan Guo, Baisong Wang, Xiao Zhang, Jian Wang, Youchun Liu, Xingdong Wang, Cheng Liu, Wenxuan Dong

**Affiliations:** 1 College of Horticulture, Shenyang Agricultural University, Shenyang 110866, China; run2010@163.com (X.W.); zhangxiao8866@syau.edu.cn (X.Z.); botelongma@163.com (J.W.); 2 Liaoning Institute of Pomology, Yingkou 115009, China; wanghongguang72@163.com (H.W.); guodan0407@163.com (D.G.); wangbaisong85@163.com (B.W.); liuyouchun911@126.com (Y.L.); wangxingdong73@163.com (X.W.)

**Keywords:** blueberry, transcriptome, proteome, cold tolerance, regulatory network

## Abstract

In China, the Liaodong Peninsula is an important growing area for blueberries because of the high organic matter content in the soil, the abundance of light, and the large temperature difference between day and night. However, the low temperature and relative humidity of the air during the winter and early spring in the Liaodong Peninsula are the main reasons for the damage to blueberry plants. Here, we documented the transcriptome and proteome dynamics in response to cold stress in three blueberry cultivars (‘Northland’, ‘Bluecrop’, and ‘Berkeley’). Functional enrichment analysis indicated that many differentially expressed genes (DEGs) and differentially abundant proteins (DAPs) were mainly involved in the pathways of protein processing in the endoplasmic reticulum, the glutathione metabolism pathway, and ribosomes. We identified 12,747 transcription factors (TFs) distributed in 20 families. Based on our findings, we speculated that cold tolerance development was caused by the expression of calcium-related genes (CDPKs and CMLs), glutathione proteins, and TFs (NAC, WRKY, and ERF). Our investigation found that three cultivars experienced cold damage when exposed to temperatures between −9 °C and −15 °C in the field. Therefore, the cold resistance of blueberries during overwintering should not only resist the influence of low temperatures but also complex environmental factors such as strong winds and low relative humidity in the air. The order of cold resistance strength in the three blueberry cultivars was ‘Berkeley’, ‘Bluecrop’, and ‘Northland’. These results provide a comprehensive profile of the response to cold stress, which has the potential to be used as a selection marker for programs to improve cold tolerance in blueberries.

## 1. Introduction

In response to cold stress caused by low temperatures, crops have obvious decreases in quality and yield during their growth process, which is a major factor that causes significant drops in agricultural production [1,2,3]. Crops respond to cold stress in various ways, including through the activation of cold resistance enzymes, reductions in water loss and photosynthesis, structural changes in cell membrane lipid fatty acids, and cell membrane disruption [4,5,6,7]. This response involves changes in gene expression, transcriptional regulation, and protein translation blockade [8]. Therefore, studying the strategies that crops use to cope with cold stress provides a powerful means for developing tools in agricultural production to prevent disasters.

Blueberries (*Vaccinium* spp.) are becoming increasingly popular because of their unique flavor and high nutritional value. Moreover, the blueberry has been described by the media as a “superfruit”, with powerful health-promoting implications for maintaining blood sugar levels, reducing oxidative stress, exerting anti-inflammatory effects, preventing cardiovascular diseases, and exhibiting antimicrobial and anticancer properties [9,10,11,12]. The Liaodong Peninsula is one of the five major blueberry-producing areas in China. In recent years, the blueberry industry has developed rapidly because of the abundant water resources and sufficient sunlight available, as well as the excellent quality and high economic value of blueberries. However, the lack of cold resistance in most of the main cultivars of blueberries is still one of the most important genetic defects [13,14,15]. Blueberries are easily affected by the low temperatures and relatively low air humidity in the winter and early spring. Different degrees of cold damage phenotypes among different cultivars, such as peeling of shoots, freezing of buds, and death of above-ground parts often occur during the overwintering process. Frost injury not only increases the workload and cost of blueberry production but also easily damages shoots during the winter protection process, resulting in lower production in the following year [16,17]. Gradually, this has become one of the main factors limiting the rapid development of the blueberry industry in northern China, especially in the Liaodong Peninsula.

Cold stress during the winter can lead to chlorosis, necrosis, and retarded growth in the germination stage. Owing to the low temperature, reactive oxygen species (ROS) and malondialdehyde levels evidently increase. These changes increase membrane rigidity, destabilize protein complexes, cause tissue browning, and damage DNA, proteins, and membrane lipids, resulting in lipid peroxidation and oxidative damage [18,19]. In order to cope with adverse conditions, plants have evolved complex antioxidant defense and osmotic regulation systems, which balance plant growth and defense responses through continuous physiological and metabolic regulation, protecting them from the destructive effects of oxidative stress [20]. Abiotic stresses induce the accumulation of PRO, sugars, organic acids, and polyols in plants and increase antioxidant enzyme activity. They remove excess ROS, protect membranes, and alleviate the osmotic stress on plants caused by environmental stress [21]. The physiological changes in blueberries during winter and early spring under field conditions in the Liaodong Peninsula are still unknown. Therefore, considering these observations and the unique conditions of the Liaodong Peninsula in the winter and early spring, it is important to analyze the cold stress mechanism in blueberries.

The cold stress response is a highly complex process that involves physiological and biochemical modifications. Cold tolerance in blueberries is controlled largely by additive gene effects and to a lesser degree by dominance gene effects [22]. Also, the carbohydrate metabolism and lipid metabolism pathways may play a vital role in blueberries during cold acclimation, and the expression of *LEA*, *CBF*, *COR78*, and *COR6.6* genes are known to increase their cold tolerance. Additionally, upstream from the production of cold-responsive proteins, TFs play a key role in the regulation of gene expression [23,24,25,26,27]. Interestingly, more transcripts were found to be upregulated under cold room conditions than under field conditions in blueberries. Many of the genes induced only under cold room conditions can be divided into stress tolerance, encode glycolytic and TCA cycle enzymes, protein synthesis. Although many similarities exist in how plants respond during cold acclimation in a cold room and in the field environment, there are major differences, suggesting caution should be taken in interpreting results based only on artificial, cold room conditions [28]. To date, many studies have been conducted in laboratories, but transcriptome and proteome studies of cold-stressed blueberries during the winter and early spring under field conditions in the Liaodong Peninsula have not yet been conducted.

Recently, many studies have combined transcriptome analysis with plant responses to biotic or abiotic stresses. This approach includes comparative transcriptome-based mining of transcription factors related to cold tolerance and screening flavonol synthesis and glutathione family genes that have regulatory effects under cold stress [29,30,31]. Glutathione is an important component of the plant antioxidant system, and its detoxification mechanism plays a crucial role in protecting cells from the accumulation of reactive oxygen species (ROS) and their reaction products [32]. Through proteomic research, it is possible to elucidate the relationship between proteins and plant stress adaptation, as well as the key biological processes and molecular foundations that affect the formation of plant stress resistance, providing an important platform for analyzing the molecular mechanisms of plant cold resistance [33,34]. Therefore, using transcriptomics and proteomics analyses to investigate the complex molecular mechanisms of blueberries during the winter and early spring under field conditions is an effective approach.

This article focused on the physiological responses and molecular characterizations of cold stress in the Liaodong Peninsula during the overwintering of blueberries. One-year-old shoots of the half-highbush blueberry cultivar ‘Northland’ (A) and the northern highbush blueberry cultivars ‘Bluecrop’ (B) and ‘Berkeley’ (C) were used as experimental materials; the shoots of these blueberry cultivars were in the dormant period. From December to March of the following year, the monthly average highest temperatures in Zhuanghe City were 1 °C, 1 °C, 2 °C, and 9 °C, respectively. The monthly average lowest temperatures were −6 °C, −7 °C, −7 °C, and 0 °C, respectively (Table S1). ‘Bluecrop’ and ‘Northland’ are the main cultivated cultivars in the field of Liaodong Peninsula, but they are severely affected by cold damage during overwintering. The cold resistance of ‘Berkeley’ was relatively strong; its cold injury was relatively light during overwintering in the Liaodong Peninsula. Under field cultivation conditions, the changes in physiological indicators such as protective enzyme activity, osmotic regulatory substance content, and active oxygen content during the overwintering period of the three cultivars were studied. The key genes and metabolic pathways that respond during overwintering were explored, the differences in the three cultivars were analyzed, and the molecular mechanisms of overwintering in the blueberries were elucidated.

## 2. Results

### 2.1. Identification of Protective Enzymes

After the early spring (cold stress), an obvious lack of fruiting (Figure S1) was found during the spring of the following year. Therefore, from 14 December 2019, to 20 March 2020 (from stage 1 to stage 7), physiological indicators in the blueberry cultivars were tested by collecting shoots at seven time points in the winter and early spring.

**Figure 1:**
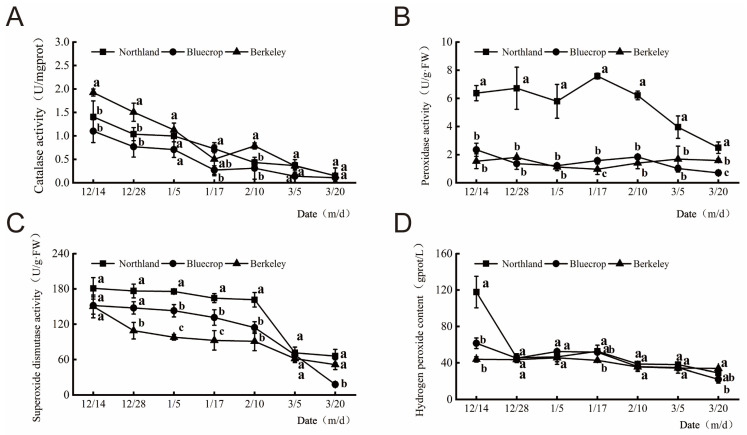
Antioxidant enzyme activity and hydrogen peroxide content in the three blueberry cultivars under winter and early spring conditions. A total of 7 stages were tested. Changes in catalase activity (**A**), peroxidase activity (**B**), superoxide dismutase activity (**C**), and hydrogen peroxide content (**D**) in the shoots of three blueberry cultivars during the overwintering period. Different small letters indicate significant differences when comparing the cultivars with each other during the same stage (Duncan’s multiple range test, *p* < 0.05, n = 3).

Catalase (CAT) (Figure 1A), peroxidase (POD) (Figure 1B), and superoxide dismutase (SOD) (Figure 1C) activity and the hydrogen peroxide content (H_2_O_2_) (Figure 1D) in the one-year-old shoots of the three cultivars were determined at different stages in this study. The activity of CAT in the shoots of the three cultivars decreased gradually during overwintering. From stage 1 to stage 7, the CAT enzyme activity in the ‘Northland’, ‘Bluecrop’, and ‘Berkeley’ cultivars was significantly reduced by 89.36%, 90.00%, and 92.23%, respectively. The POD activity in ‘Northland’ shoots decreased gradually during the overwintering period, while the POD activity in ‘Bluecrop’ and ‘Berkeley’ shoots changed relatively slightly during the overwintering period. In stage 1 and stage 6, there was no significant difference in SOD activity among the shoots of the three cultivars. In stage 2 and stage 7, the SOD activity in the ‘Northland’ and ‘Bluecrop’ shoots was significantly higher than that in ‘Berkeley’ shoots. In stage 3 and stage 4, the SOD activity in the three cultivars had obvious differences and decreased in the order of ‘Northland’, ‘Bluecrop’, and ‘Berkeley’. The contents of H_2_O_2_ in the three cultivars showed a trend of decreasing first, then increasing, and then decreasing again.

### 2.2. Measurement of Osmotic Regulatory Substances

**Figure 2:**
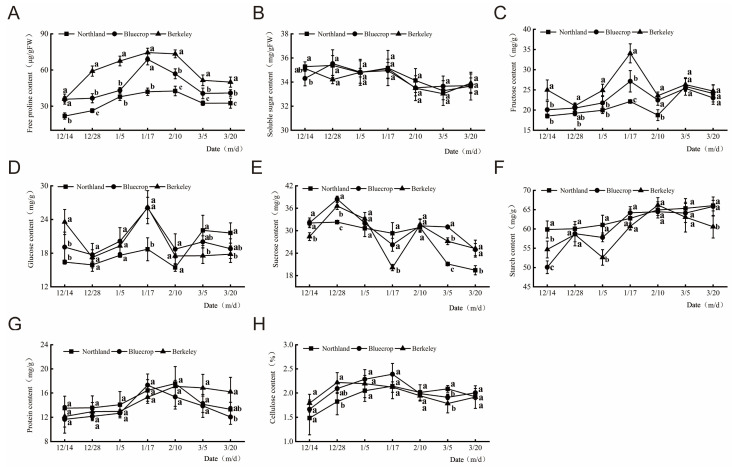
Osmotic regulating substance content in the three blueberry cultivars under winter and early spring conditions. A total of 7 stages were tested. Changes in free proline content (**A**), soluble sugar content (**B**), fructose content (**C**), glucose content (**D**), sucrose content (**E**), starch content (**F**), protein content (**G**), and cellulose content (**H**) in the shoots of the three blueberry cultivars during the overwintering period. Different small letters indicate significant differences when comparing the cultivars with each other during the same stage (Duncan’s multiple range test, *p* < 0.05, n = 3).

The contents of osmotic regulatory substances, including the free proline content, soluble sugar content, fructose content, glucose content, sucrose content, starch content, protein content, and cellulose content (Figure 2), were measured in our study. Within the sampling period, the free proline content in the three cultivars showed a “single peak” trend over time, but the peak periods in the three cultivars were different. In stage 1, the contents of free proline in the shoots of the ‘Northland’, ‘Bluecrop’, and ‘Berkeley’ cultivars were 21.83, 35.78, and 35.93 μg/g (fresh weight, FW), respectively (Figure 2A). The total soluble sugar content in the three cultivars showed an “S”-shaped change over time. ‘Northland’ and ‘Bluecrop’ had the highest total soluble sugar contents in stage 2, which were 35.38 and 35.53 mg/g (FW), respectively (Figure 2B). ‘Berkeley’ had the highest total soluble sugar content (35.12 mg/g (FW)) in stage 1. The fructose content in the three cultivars showed a “single peak” trend over time, and the peak periods in the three cultivars were different. In stage 1, the fructose contents in the shoots of ‘Northland’, ‘Bluecrop’, and ‘Berkeley’ were 18.55, 20.10, and 24.99 mg/g, respectively (Figure 2C). The glucose content in the three cultivars showed a “single peak” trend over time, and the peak periods in the three cultivars were different. In stage 1, the glucose contents in shoots of the cultivars ‘Northland’, ‘Bluecrop’, and ‘Berkeley’ were 16.42, 19.08, and 23.56 mg/g, respectively. The glucose content in both the ‘Bluecrop’ and ‘Berkeley’ shoots peaked in stage 4, at 26.08 and 26.19 mg/g, respectively (Figure 2D). The glucose content in ‘Northland’ shoots peaked at 22.07 mg/g (FW) in stage 6.

The sucrose content of the three cultivars showed a decreasing trend with time. In stage 1, the sucrose contents in the ‘Northland’, ‘Bluecrop’, and ‘Berkeley’ shoots were 32.07, 32.12, and 28.43 mg/g (FW), respectively. The sucrose content in the ‘Berkeley’ shoots was lowest in stage 4, at 20.17 mg/g (FW). The sucrose contents in both the ‘Northland’ and ‘Bluecrop’ shoots were lowest in stage 7, at 19.44 and 24.96, respectively (Figure 2E). The starch content showed an “S”-shaped change over time. In stage 1, the starch contents in the ‘Northland’, ‘Bluecrop’, and ‘Berkeley’ shoots were 59.87, 50.08, and 54.69 mg/g (FW), respectively. In stage 7, the starch content in the ‘Northland’, ‘Bluecrop’, and ‘Berkeley’ cultivars increased to 66.06, 65.83, and 60.58 mg/g (FW), respectively (Figure 2F). The protein content in the three cultivars showed a “single peak” trend over time, but the peak periods in the three cultivars were different. In stage 1, the protein contents in ‘Northland’, ‘Bluecrop’, and ‘Berkeley’ shoots were 13.51, 11.66, and 12.14 mg/g (FW), respectively (Figure 2G). The cellulose content in the shoots first increased and then decreased over time. In stage 1, the cellulose contents in the ‘Northland’, ‘Bluecrop’, and ‘Berkeley’ shoots were 1.48%, 1.67%, and 1.79%, respectively. ‘Northland’ and ‘Bluecrop’ had the highest cellulose contents in stage 4, at 2.14% and 2.39%, respectively (Figure 2H). ‘Berkeley’ had the highest cellulose content in its shoots in stage 2, at 2.22%.

### 2.3. Establishing the Transcriptomes and Proteomes of the Three Blueberry Cultivars under Cold Stress

In order to improve the analysis of gene expression changes and differences, on 14 December 2019 (stage 1), 28 December 2019 (stage 2), 17 January 2020 (stage 4), 10 February 2020 (stage 5), and 5 March 2020 (stage 6), the transcriptomes in the blueberry cultivars were sequenced by collecting shoots at five time points in the winter and early spring. An average of 1.86 Gb of clean data was obtained from each sample. Through comparison to the reference genome with Minimap2 software (version 2.16) and redundancy analysis [35], 160,176 transcripts were obtained, of which 31,617 transcripts were newly identified. Moreover, 1393 lncRNAs were identified, and 25,762 new transcripts were functionally annotated. The quality control results are shown in Table S2.

In order to improve the analysis of protein changes and differences, on 14 December 2019 (stage 1), 28 December 2019 (stage 2), 17 January 2020 (stage 4), and 10 February 2020 (stage 5), the proteomes in the blueberry cultivars were sequenced by collecting shoots at four time points in the winter and early spring. The analysis of the proteome for samples used Label-Free Liquid Chromatography–Tandem Mass Spectrometry (LC-MS/MS). In total, 8351 proteins were identified, of which 5852 proteins were annotated, including 5368 in the GO annotation database and 2360 in the KEGG annotation database. PCA indicated that the three biological replicates of the ‘Northland’ (A), ‘Bluecrop’ (B), and ‘Berkeley’ (C) cultivars had good conformity. Overall, all biological replicates showed high overlap in the proteome (Figure S2A) and transcriptome (Figure S2B).

### 2.4. Differentially Expressed Gene and Protein Sets in the Three Blueberry Cultivars

We identified gene sets showing significant differential expression in the three cultivars in each stage of cold stress (Table 1). A total of 28724 genes exhibited significantly differential expression in different comparison groups (Table S3). The largest number of DEGs between the two cultivars (10,676 in A6 vs. C6) exhibited differential expression in stage 6. Moreover, 10,448, 10,428, and 10,082 genes were differentially expressed in A5 vs. B5, A4 vs. B4, and A2 vs. B2, respectively. In the comparison of ‘Bluecrop’ and ‘Berkeley’, the numbers of up- and downregulated genes were roughly equal. For instance, 3850 and 3760 DEGs were up- and downregulated, respectively, in B1 vs. C1. However, the number of DEGs between different stages in the same cultivars observably decreased. Compared with the A1 stage, 193, 1616, 1566, and 1263 DEGs were identified in the A2, A4, A5, and A6 stages, respectively. By comparing different stages in ‘Bluecrop’, 620, 1307, 2686, and 2579 DEGs were identified in B1 vs. B2, B1 vs. B4, B1 vs. B5, and B1 vs. B6, respectively. Moreover, compared with the C1 stage, 282, 928, 3080, and 5004 DEGs were identified in the C2, C4, C5, and C6 stages, respectively.

A total of 8351 proteins were identified by proteome analysis (Table S4), and DEPs were also identified. A comparison of DEPs at the different time points relative to the control (2 vs. 1, 4 vs. 1, and 5 vs. 1) revealed 298, 82, and 84 DEPs with significantly altered expression levels in the ‘Northland’ (A), ‘Bluecrop’ (B), and ‘Berkeley’ (C) cultivars, respectively (Figures S3 and S4). Furthermore, more DEPs were identified in different cultivars. In stage 1, 979 DEPs were identified, among which 17 common DEPs were found. A total of 864 DEPs were found in the three cultivars by stage 2. By pairwise comparison, in stage 4, 717 DEPs, including 12 common DEPs, were found. Moreover, we found 872 DEPs in stage 5 by pairwise comparisons of the three cultivars. Subsequently, the functions of the DEPs were annotated by KEGG enrichment analysis. Interestingly, many DEGs were enriched in the glutathione metabolism pathway.

Evidently, the numbers of DEPs and DEGs among the different cultivars were greater than those in the same cultivars at different time points. We speculated that the DEPs and DEGs among the different cultivars were the main reason for the difference in cold hardiness.

### 2.5. Expression Pattern and Functional Enrichment Analysis of the DEGs

**Figure 3:**
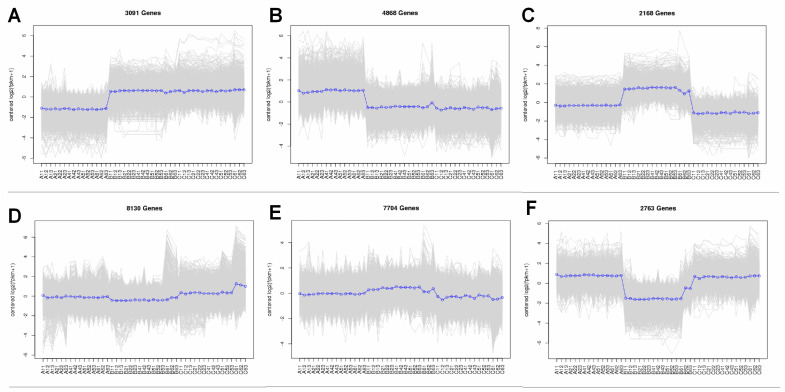
Expression trends in genes in six clusters (**A**–**F**) during the four developmental stages. The lines represent the expression levels of individual genes. The thick dotted lines represent the average expression levels of the genes in the cluster. The number of genes in each cluster is indicated.

In addition, K-means cluster analysis was performed, and all DEGs were clustered into six groups, as shown in Figure 3. This analysis showed the gene expression changes under cold stresses in all samples. According to the gene expression trends, 3091 genes (subcluster 1) displayed higher expression in the ‘Bluecrop’ and ‘Berkeley’ cultivars (Figure 3A). The results of subcluster 2 indicated that 4868 genes were more highly expressed in the ‘Northland’ cultivar (Figure 3B). The 2168 genes in subcluster 3 were evidently more highly expressed in the ‘Bluecrop’ cultivar (Figure 3C). The results of subcluster 4 indicated that 8130 genes were more highly expressed in the ‘Northland’ and ‘Berkeley’ cultivars (Figure 3D). The 7704 genes in subcluster 5 were evidently more highly expressed in the ‘Bluecrop’ cultivar (Figure 3E). In subcluster 6, 2763 genes were expressed at lower levels in the ‘Bluecrop’ cultivar (Figure 3F). These results indicate apparent differences among cultivars in the responses of the genes and pathways to cold stress.

**Figure 4:**
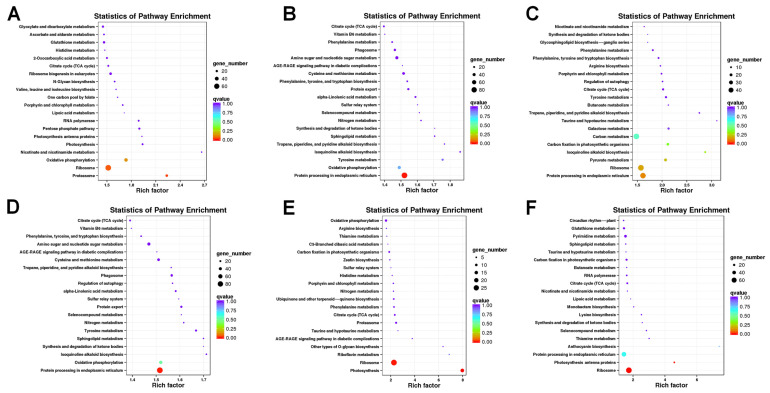
KEGG enrichment analysis of the genes in the six clusters (**A**–**F**). The DEGs were enriched in the ribosome, proteasome, protein processing in the endoplasmic reticulum, photosynthesis, and photosynthesis antenna protein pathways.

To determine the functional significance of the transcriptional changes in each cultivar, KEGG enrichment analysis was performed with the genes of the six subclusters (Figure 4). In subcluster 1, the DEGs were enriched in the ribosome and proteasome pathways (Figure 4A). In subclusters 2 and 4, the DEGs were enriched in the protein processing in the endoplasmic reticulum pathway (Figure 4B,D). The DEGs in subcluster 3 were enriched in the protein processing in the endoplasmic reticulum and ribosome pathways (Figure 4C). The DEGs were enriched in the photosynthesis and ribosome pathways in subcluster 5 (Figure 4E). The results of subcluster 6 indicated that the DEGs were enriched in the photosynthesis antenna protein and ribosome pathways (Figure 4F). These pathways are functionally relevant to this study and may play an important role in cold resistance. Further analysis showed that differentially expressed genes were significantly enriched in pathways such as ascorbic acid and aldehyde metabolism, glutathione metabolism, citrate cycle (TCA cycle), and glycolysis/gluconeogenesis.

### 2.6. Alternative Splicing and lncRNA Analysis

**Figure 5:**
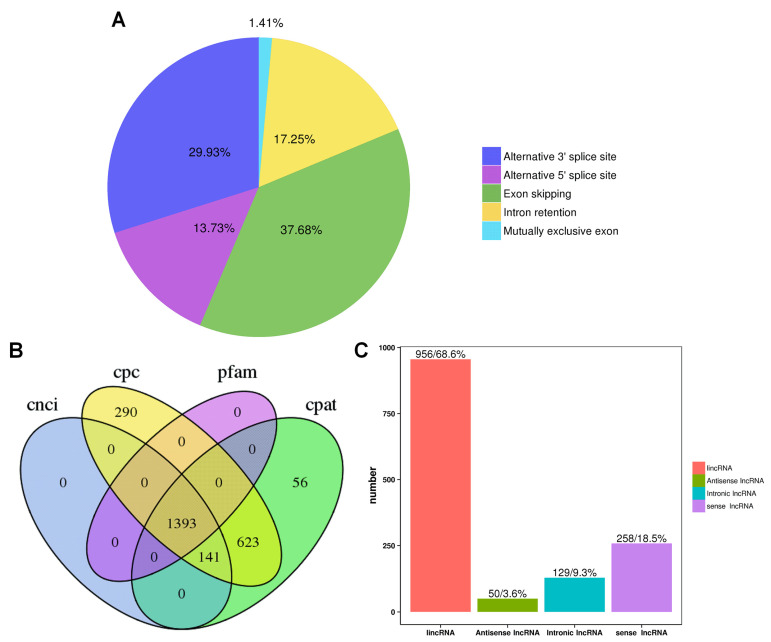
Alternative splicing and lncRNA analysis. (**A**) Classification of alternative splicing events and the proportions of each type of alternative splicing. (**B**) The numbers of lncRNAs determined by four software analysis programs. (**C**) Proportions of the four kinds of lncRNAs classified.

Alternative splicing can result in the splicing of precursor mRNA to produce different mature RNAs that can be translated into related proteins representing the diversity of biological traits. Our study found that the full-length transcripts were statistically categorized into transcripts undergoing five alternative splicing events (Figure 5A). Among these five alternative splicing events, exon skipping (37.68%) was the most prominent, followed by alternative 3’ splice site (29.93%), intron retention (17.25%), alternative 5’ splice site (13.73%), and mutually exclusive exon (1.41%) events, similar to the findings of previous reports on rose in response to stress [36].

Regulatory functions are one of the main functions of lncRNAs and are vital for posttranscriptional, transcriptional, and epigenetic regulation. CPC analysis, CNCI analysis, CPAT, and Pfam protein domain analysis were used to predict lncRNAs from transcriptome data. A total of 1393 lncRNAs were predicted by CPC analysis, CNCI analysis, CPAT analysis, and Pfam software (version v1.3, Figure 5B). Subsequently, the lncRNAs were classified and mapped according to the reference genome annotation information. The results indicated that 956 (68.6%) of the lncRNAs were classified as lincRNA (long intergenic noncoding RNA; and intergenic lncRNAs were compared), and only 50 (3.6%) of the lncRNAs were classified as antisense lncRNAs (lncRNA as). Intronic lncRNAs and sense lncRNAs accounted for 9.3% (129) and 18.5%, respectively (Figure 5C).

### 2.7. Transcription Factor-Related DEGs

**Figure 6:**
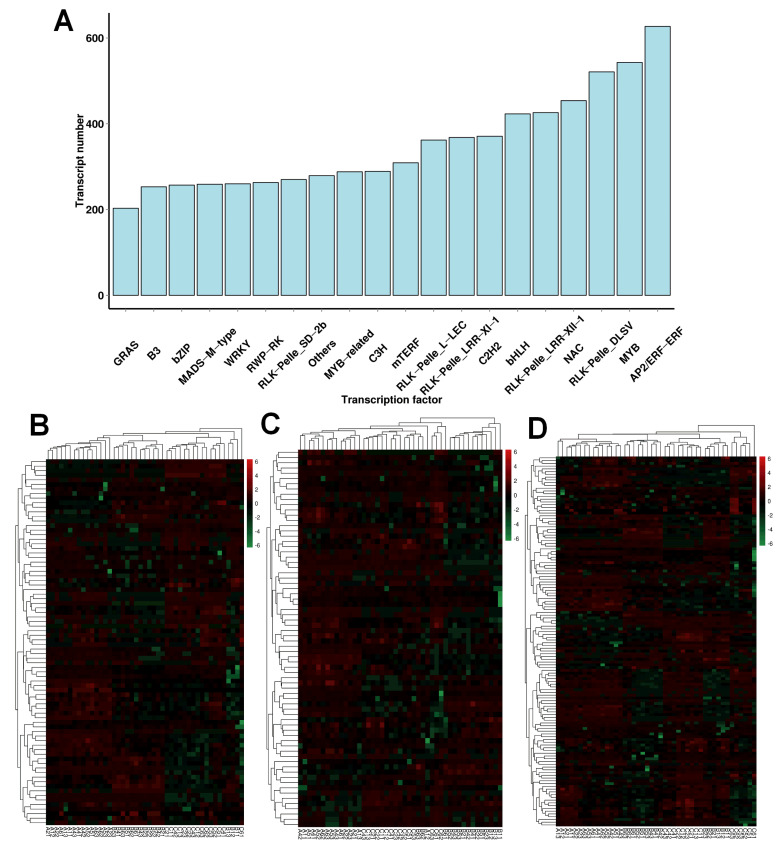
The number of TFs. (**A**) A total of 12747 TFs were distributed in 20 families. Heatmaps of the relative expression levels of NAC (**B**), WRKY (**C**), and ERF (**D**).

Given the important regulatory function of transcription factors (TFs), TF-encoding genes were identified by performing a search of the Plant Transcription Factor Database (PlantTFDB, http://planttfdb.gao-lab.org/, accessed on 10 March 2021). We identified 12747 TFs distributed in 20 families (Table S4, Figure 6A). These TFs were mainly in the following families: AP2/ERF-ERF (612 genes), WRKY (237 genes), bHLH (227 genes), MYB (581 genes), NAC (560 genes), RLK-Pelle_DLSV (543 genes), and RLK-Pelle_LRK10L-2 (468 genes). More details of the expression profiles of the identified TFs are provided in Table S5. NAC, WRKY, and ERF play essential roles in the response to cold stress, and we focused on these three TFs. A total of 80 NAC family TFs (Table S6) were identified and differentially expressed, and the heatmap shows the expression trends in these genes. Moreover, 72 WRKY family TFs (Table S7) and 134 ERF family TFs (Table S8) were identified by using a heatmap (Figure 6B–D).

### 2.8. Calcium-Related Gene Expression in the Three Cultivars

**Figure 7:**
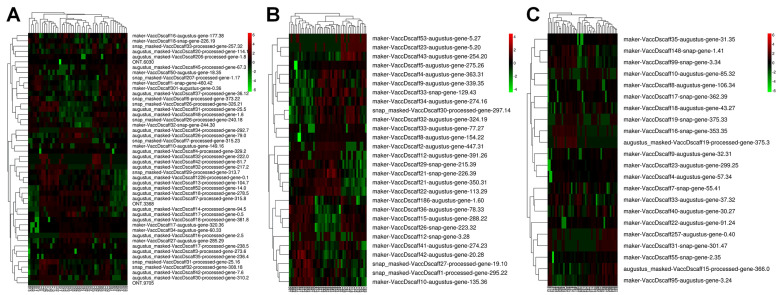
A total of 106 calcium-related DEGs were identified. Heatmaps of the DEGs in the calcium ion binding (GO:0005509) (**A**), calcium-dependent cysteine-type endopeptidase activity (GO:0004198) (**B**), and calcium-transporting ATPase activity (GO:0005388) terms (**C**).

A total of 106 calcium-related DEGs were identified in our research (Table S9). Forty-eight DEGs were classified as being involved in calcium ion binding (GO:0005509) (Figure 7A), including the genes encoding calcium-binding protein CML27, calcium-binding protein CML13, calcium-binding protein CML19, calcium-binding protein CML11, and calcium-binding protein CML42. Eight calcium-binding proteins, including CML50, CML49, and CML48, were identified and classified as being involved in calcium-dependent cysteine-type endopeptidase activity (GO:0004198) (Figure 7B). In the calcium-transporting ATPase activity term (GO:0005388) (Figure 7C), 22 cation-transporting ATPase genes were found, including calcium-transporting ATPase 1, calcium-transporting ATPase 3, calcium-transporting ATPase 9, calcium-transporting ATPase 13, and calcium-transporting ATPase 4. In calmodulin-dependent protein kinase activity term (GO:0004683), 28 calcium-dependent protein kinases (CDPKs) were identified. Evidently, the CDPKs were differentially expressed among the three cultivars. For example, snap_masked-VaccDscaff1-processed-gene-295.22, maker-VaccDscaff10-augustus-gene-135.36, and snap_masked-VaccDscaff27-processed-gene-19.10 were more highly expressed in the ‘Bluecrop’ cultivar. In addition, maker-VaccDscaff53-augustus-gene-5.27, maker-VaccDscaff23-augustus-gene-5.20, maker-VaccDscaff43-augustus-gene-254.20, and maker-VaccDscaff5-augustus-gene-275.26 had higher expression in the ‘Berkeley’ cultivar.

### 2.9. Restructuring of the Proteome in Response to Cold Stress

**Figure 8:**
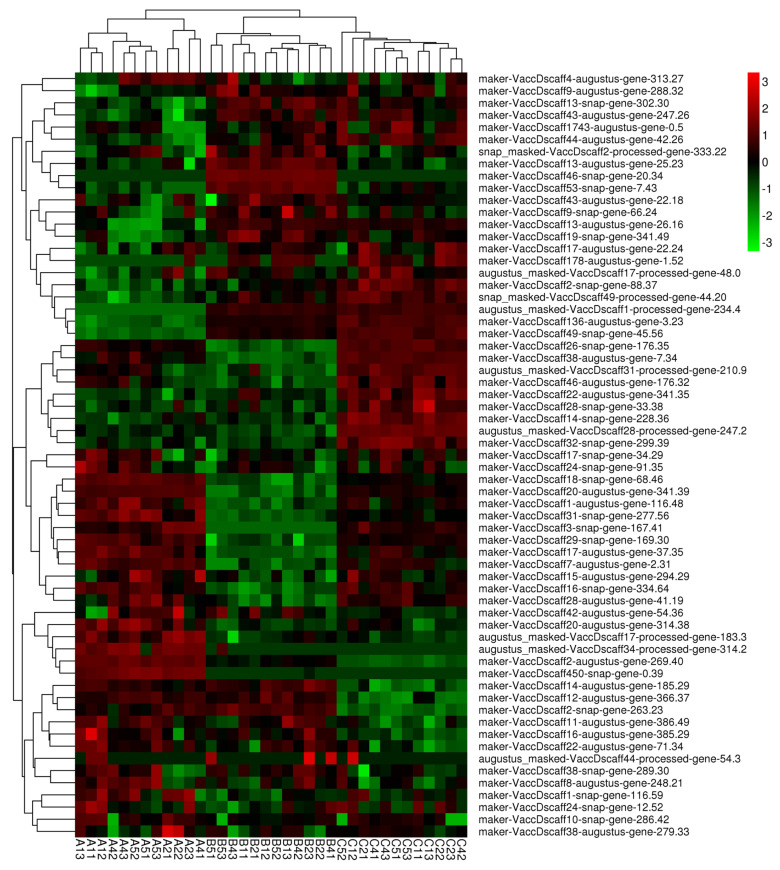
Heatmap diagram of the relative expression levels of glutathione proteins.

The analysis showed that differentially expressed proteins were significantly enriched in pathways such as glutathione metabolism, the pentose phosphate pathway, biosynthesis of amino acids, ascorbate and aldarate metabolism, other glycan degradation, and amino sugar and nucleotide sugar metabolism. We focused on the difference in glutathione protein expression among the three cultivars in response to cold stress. Sixty-three glutathione proteins displayed significantly different expression levels in response to cold stress in the three cultivars (Table S10). Evidently, the proteins were not differentially expressed among the different stages in each cultivar but were differentially expressed among the three cultivars (Figure 8). Many proteins, including maker-VaccDscaff13-augustus-gene-25.23, maker-VaccDscaff46-snap-gene-20.34, maker-VaccDscaff53-snap-gene-7.43, and maker-VaccDscaff43-augustus-gene-22.18, were expressed at higher levels in the ‘Bluecrop’ cultivar than in the ‘Northland’ and ‘Berkeley’ cultivars. In addition, augustus_masked-VaccDscaff34-processed-gene-314.2, augustus_masked-VaccDscaff17-processed-gene-183.3, maker-VaccDscaff2-augustus-gene-269.40, and maker-VaccDscaff450-snap-gene-0.39 were more highly expressed in the ‘Northland’ cultivar. Moreover, maker-VaccDscaff46-augustus-gene-176.32, maker-VaccDscaff22-augustus-gene-341.35, maker-VaccDscaff28-snap-gene-33.38, and maker-VaccDscaff14-snap-gene-228.36 were specific to the ‘Berkeley’ cultivar. Taken together, these findings indicated that glutathione proteins play an important role in the response to cold stress.

### 2.10. Real-Time PCR and the Correlation Analysis between Transcriptomes and Proteomes

**Figure 9:**
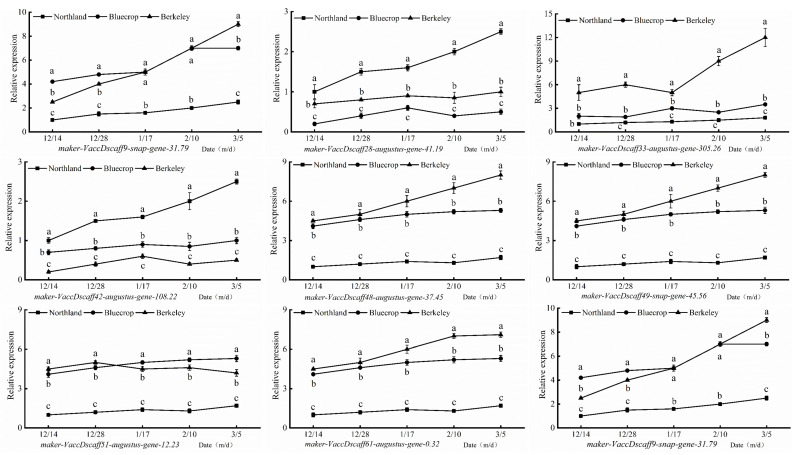
Validation of 9 selected DEGs via qRT-PCR. VcUBC28 was used as an internal control. The values are means (±) SDs of three independent biological repeats. Different small letters indicate significant differences when comparing the cultivars with each other during the same stage (Duncan’s multiple range test, *p* < 0.05, n = 3).

The real-time PCR results show that the change trends in the nine genes were basically consistent with the transcriptome results, indicating that the transcriptome sequencing results were accurate and reliable (Figure 9).

**Figure 10:**
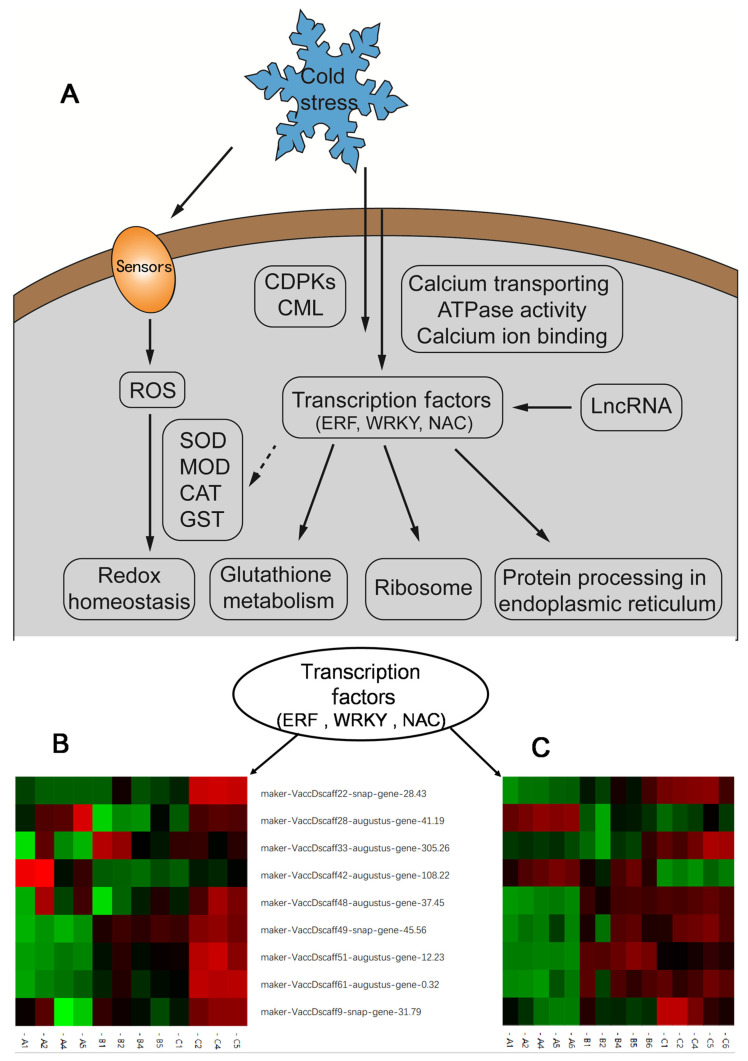
Schematic of the molecular mechanism for the response to cold in three blueberry cultivars. (**A**) We integrated the potential pathways and genes involved in the difference in cold resistance. (**B**) Intergroup clustering heatmap of DEPs. (**C**) Intergroup clustering heatmap of DEGs. Red indicates upregulation of DEPs and DEGs expression, while green indicates downregulation of DEPs and DEGs expression(**B**,**C**).

The conducted inter group clustering heatmaps on differentially expressed genes and proteins related to transcription factors (ERF, WRKY, NAC). The expression patterns of differential genes and differential proteins were similar, indicating that transcriptome was highly correlated with proteome. Thus, we speculated that the screened genes and proteins may play an important role in the cold resistance mechanism (Figure 10).

## 3. Discussion

During rapid climate change, cold is considered a destructive pressure based on its consequences. Temperature plays a key role in the growth and production of blueberries. We explored the differences in transcript and protein responses to cold stress in three cultivars in different stages.

Previous research found that plants adapt to environmental stresses by different mechanisms, including changes in morphological patterns and physiological and biochemical processes [18]. Various abiotic stresses often result in the excessive accumulation of ROS in plants and inflammatory damage to various cellular structures [37]. To maintain the dynamic balance of oxidation and reduction in plants, the effective activity of antioxidant enzymes such as CAT and SOD can eliminate excessive ROS [38]. Interestingly, the activity of catalase (CAT), peroxidase (POD), and superoxide dismutase (SOD) in the shoots of the three cultivars decreased gradually during overwintering. Thus, we speculated that these enzymes might be involved in the response to cold stress. Cold stress adaptation is associated with the maintenance of osmotic homeostasis by metabolic adjustments, leading to the accumulation of metabolically compatible compounds such as soluble sugars and proline. Thus, in the present study, we determined the contents of osmotic regulatory substances, including the free proline content, soluble sugar content, fructose content, sucrose content, starch content, protein content, and cellulose content, under low-temperature stress. The free proline content in the three cultivars first increased and then decreased. In stage 1, the contents of free proline in shoots of the ‘Northland’, ‘Bluecrop’, and ‘Berkeley’ cultivars were 21.83, 35.78, and 35.93 μg/g fresh weight, respectively. The protein content in the three cultivars showed a “single peak” trend over time, but the peak periods in the three cultivars were different. In stage 1, the protein contents in the ‘Northland’, ‘Bluecrop’, and ‘Berkeley’ shoots were 13.51, 11.66, and 12.14 mg/g, respectively. The accumulation of proline can greatly enhance the abiotic stress resistance capability of plants [39]. From December 14th, 2019 to March 20th, 2020 (from stage 1 to stage 7), The CAT activity, PRO, and sucrose content of the one-year-old ‘Berkeley’ shoots were generally higher than those in the ‘Northland’ and ‘Bluecrop’ shoots. Before February 10th, the fructose and glucose content in the one-year-old ‘Berkeley’ shoots were higher than those in the ‘Northland’ and ‘Bluecrop’ shoots.

The Omics era has brought a newfound understanding of the tenets that underlie genome organization and transcriptional control [40]. Many studies have shown that transcription factors (TFs), such as NAC, AP2/ERF, and WRKY, play an important role in abiotic stress responses [41,42,43]. Several WRKY genes have been found in plants owing to their central roles in biotic and abiotic stress responses [44]. A close relationship between WRKY family members and antioxidant-related genes has been described. The overexpression of OsWRKY76 in rice increased the expression of antioxidant-related genes, such as peroxidases (OsPrx16/17, OsPrx39, and OsPrx74) and GSTs (OsGSTU5), thus improving cold tolerance [45]. The overexpression of VaWRKY12 enhanced cold tolerance in transformed *Arabidopsis* and grapevine seedlings [46]. In our study, a total of 237 WRKYs, including 72 differentially expressed WRKYs, were identified by screening, indicating the importance of WRKY genes in the response to cold stress. Studies on NACs have shown that they are involved in different biological processes, including plant development, secondary growth, and responses to different plant species under abiotic stress [47,48]. The NAC transcription factor *CaNAC064* is a regulator of cold stress tolerance in peppers. Low temperature induces the generation of ROS, including H_2_O_2_ and O^2−^, which leads to oxidative stress in peppers with reduced expression of *CaNAC064*. Oxidative stress can cause oxidative damage; the NAC protein plays an important role in the regulation of antioxidant genes under stress conditions, which could increase stress tolerance by reducing the accumulation of ROS [49]. A report indicated that the overexpression of *CaNAC064* reduced oxidative damage by maintaining high SOD, POD, and CAT activities, thereby reducing the accumulation of ROS, which in turn conferred a higher cold tolerance on the transgenic lines [47]. This also explains the differential expression of these transcription factors observed in this study.

Calcium (Ca^2+^) is an important secondary messenger in plant cells and has been found to be involved in the response and adaptation of plants to external stresses [50]. In plant cells, the level of Ca on the cell membrane increases significantly in response to various abiotic stresses. As an important mediator, calcium can participate in the perception and transmission of various stress signals through its downstream calcium-binding proteins, thus triggering a series of biochemical reactions to adapt to or resist various stresses. A study demonstrated that Ca^2+^ plays an important role in improving cold resistance in plants and maintaining the stability of the membrane system [51]. Calcium-dependent protein kinases (CDPKs) participate in the intermediate process instead of functioning in the initial response to cold stress. Moreover, several Ca^2+^-related genes, such as CDPK13, are regulated by low-temperature stress in plants. A rice line with CDPK13 overexpression exhibited stronger cold tolerance and a higher rate of plant recovery from cold stress, indicating that CDPK13 may be a central protein in the cold stress response signaling network in rice [52]. A total of 106 calcium-related genes were identified and found to be differentially expressed; these genes included those encoding calcium-binding protein CML27, calcium-binding protein CML13, calcium-binding protein CML19, calcium-binding protein CML11, and calcium-binding protein CML42. Specifically, 28 calcium-dependent protein kinases (CDPKs) were differentially expressed among the three cultivars. We speculated that CDPKs might be key proteins in the response to low-temperature stress. In addition, maker-VaccDscaff53-augustus-gene-5.27, maker-VaccDscaff23-augustus-gene-5.20, maker-VaccDscaff43-augustus-gene-254.20, and maker-VaccDscaff5-augustus-gene-275.26 had higher expression in the ‘Berkeley’ cultivar, which are the reasons why the ‘Berkeley’ cultivar has stronger cold resistance than the other two cultivars.

Amino acid metabolism plays an important role in plant signal transduction and osmotic regulation [53]. Carbohydrates can provide basic energy for plants under adversity to maintain normal life activities and adapt to harsh environments [54,55]. In this study, it was found that differentially expressed genes and proteins were significantly enriched in pathways such as amino acid metabolism and carbohydrate metabolism. They can regulate the content of osmoregulatory substances such as proline and glucose to promote stress resistance in blueberries. Glutathione (GSH), a master antioxidant, plays a significant role in the stress defense process in plants. Lowering the content of cellular GSH has harmful effects on the defense mechanisms of plants against environmental stress [56,57,58]. A study indicated the role of GSH in modulating the levels of various protein repertoires, including MPKs, under different stresses [59]. A stress-induced increase in cellular GSH was found to modulate MPK3 expression positively via WRKY40-mediated transcriptional upregulation. Sixty-three glutathione proteins displayed significantly different expression levels in response to cold stress among the three cultivars. We found maker-VaccDscaff46-augustus-gene-176.32, maker-VaccDscaff22-augustus-gene-341.35, maker-VaccDscaff28-snap-gene-33.38, and maker-VaccDscaff14-snap-gene-228.36 were specific to the ‘Berkeley’ cultivar. These glutathione proteins play an important role in the response to cold stress, which may be the reason why the ‘Berkeley’ cultivar has a stronger cold resistance than the ‘Northland’ and ‘Bluecrop’ cultivars. In our research, which combined transcriptomics with proteomic analysis, a cold stress-responsive molecular network was constructed with most of the significantly enriched cold-responsive genes and proteins in blueberries (Figure 10). When blueberry plants are exposed to cold stress, some plasma membrane-located sensors mobilize signals such as Ca^2+^ and ROS. Following the transduction of these signals, the related protein kinases, CDPKs and CML, are activated to interact with TFs and trigger the expression of downstream cold-tolerant genes. This process leads to the production of a large number of proteins, which explains the protein processing activity in the endoplasmic reticulum and ribosome pathways. Persistent cold stress disrupts the balance between ROS production and scavenging in plant cells. To respond to oxidative damage, plants strive to maintain cellular redox homeostasis by increasing the biosynthesis and activity of antioxidant enzymes, including GST, CAT, and SOD, and the content of osmoregulation substances. Under cold stress, plants cope by increasing the expression of the key protein in glutathione metabolism, GSH. The molecular network covered many functional components, including TF regulation (NAC, WRKY, and ERF), protein processing in the endoplasmic reticulum, glutathione metabolism, and calcium-related gene expression, allowing us to further understand the principle of cold tolerance. In addition, some of these molecules can be used as selection markers in cold tolerance programs in blueberries. Moreover, alternative splicing and lncRNAs also play important roles in the regulation of gene expression.

## 4. Materials and Methods

### 4.1. Experimental Site and Plant Materials

Field experiments were conducted for 2 years in 2019 and 2020 at the blueberry base of the Dalian Hongjia Blueberry Cooperative in Zhuanghe City, Liaoning Province (E123°05′81″, N39°88′29″). From December to March of the following year, the monthly average highest temperatures in Zhuanghe City were 1 °C, 1 °C, 2 °C, and 9 °C, respectively. The monthly average lowest temperatures were −6 °C, −7 °C, −7 °C, and 0 °C, respectively. The precipitation was 19.9, 0.3, 24.9, and 4.7 mm, respectively; and the minimum temperature was −15 °C (on 5 and 6 February) (weather network: http://lishi.tianqi.com, accessed on 3 March 2021, Table S1). One-year-old shoots of ‘Northland’ (A), ‘Bluecrop’ (B), and ‘Berkeley’ (C) cultivars were collected at seven time points in the winter and early spring. The one-year-old shoots of the blueberry cultivars used in the experiments were in the dormant period, and the samples were stored at −80 °C until RNA or protein extraction. In total, 15 groups and 45 samples were used for transcriptome analysis, and 12 groups and 36 samples were used for proteome analysis, with three biological replicates for each group. Detailed information is presented in Table S11.

### 4.2. RNA Isolation and RNA-Seq Library Construction

For cDNA library construction and sequencing analysis, total RNA from the one-year-old blueberry shoots was extracted from all the samples. The cDNA-PCR Sequencing Kit (SQK-PCS109) protocol provided by Oxford Nanopore Technologies (ONT, Nanopore, Oxford, UK) was utilized to prepare total RNA for cDNA libraries [60]. Briefly, the template-switching activity of reverse transcriptase was exploited to enrich full-length cDNAs and add defined PCR adapters directly to both ends of the first-strand cDNA. Then, LongAmp Taq (NEB) (New England Biolabs, Ipswich, MA, USA) was used to perform PCR amplification of the cDNA for 14 cycles. The PCR products were subjected to ONT adaptor ligation with T4 DNA ligase (M0202M, NEB) (New England Biolabs, Ipswich, MA, USA). According to the ONT protocol, Agencourt XP beads were utilized to purify the DNA. The final cDNA libraries were loaded into FLO-MIN109 flow cells, and the PromethION platform of Biomarker Technology Company (Beijing, China) was used to run the sequencing program.

### 4.3. Transcriptome Assembly and Gene Annotation

After RNA sequencing, the raw reads with a minimum average read quality score of 7 bp and a minimum read length of 500 bp were filtered. The selected reads were mapped to the rRNA database, and ribosomal RNAs were discarded. Then, both ends of the reads were searched for primers to identify the full-length, nonchimaeric (FLNC) transcripts. After mapping to the reference genome with Minimap2 (version 2.16, https://github.com/lh3/minimap2, accessed on 12 March 2021), we obtained the clusters of the FLNC transcripts. After refining the data within each cluster with pinfish, the consensus isoforms were obtained. Minimap2 was employed to map the consensus sequences to the reference genome [61]. The cDNA Cupcake package (https://github.com/Magdoll/cDNA_Cupcake, accessed on 12 March 2021) was used to further fold the mapped reads with a minimum coverage of 85% and a minimum identity of 90%. When collapsing redundant transcripts, we did not consider differences in the 5’ end. iTAK was applied to identify the plant transcription factors [62]. To sort non-protein-coding RNA candidates from putative protein-coding RNAs in the set of transcripts, the following four computational approaches were combined: CPC (https://github.com/gao-lab/CPC2_standalone, accessed on 12 March 2021), CNCI (https://github.com/www-bioinfo-org/CNCI, accessed on 12 March 2021), CPAT (https://rna-cpat.sourceforge.net/, accessed on 12 March 2021) and Pfam (http://pfam.xfam.org/ accessed on 12 March 2021).

The R package DESeq2 (1.6.3) was employed to perform differential expression analysis between two conditions/groups [63]. The Benjamini–Hochberg procedure for controlling the false discovery rate was used to calculate the *p* values. The adjusted *p* value (*p* < 0.01) and fold change ≥ 1.5 were defined as the criteria for identifying significantly differentially expressed genes (DEGs). Next, selected DEGs were mapped to the KEGG (Kyoto Encyclopedia of Genes and Genomes) pathway database for pathway enrichment analysis [64,65,66]; they were similarly mapped to GO (Gene Ontology) terms for functional GO enrichment analysis in the following 3 GO categories: molecular function, cellular component, and biological process. Gene function was annotated based on the following databases: NR (NCBI nonredundant protein sequences), Pfam (Protein family), KOG/COG/eggNOG (Clusters of Orthologous Groups of proteins), and Swiss-Prot (a manually annotated and reviewed protein sequence database). These data can be found in the National Center for Biotechnology Information (NCBI) BioProject database under accession number PRJNA 910836.

### 4.4. Label-Free Liquid Chromatography–Tandem Mass Spectrometry (LC–MS/MS)

Samples stored at −80 °C were ground into a fine powder, and lysis buffer was then used to extract proteins. Sample preparation for label-free LC-MS/MS was carried out according to [67,68]. Briefly, 5 µg of total protein was subjected to 1-DE to remove nonprotein compounds. Then, trypsin (trypsin–protein = 1:25) was added, and the samples were incubated at 37 °C overnight. The resulting peptides were purified using solid-phase extraction (AMR, Tokyo, Japan).

For peptide separation, an ADVANCE UHPLC system (Michrom Bioresources, Auburn, CA, USA) was used [69]. Peptide solutions were reconstituted with 20 µL of 2% methanol and 0.1% formic acid. The solutions were centrifuged at 12,000 rpm for 10 min, and the supernatant was aspirated for loading. The loaded volume was 10 μL, and the sandwich loading method was used. The loading pump flow rate was 300 nL/min over 15 min, while the separation flow rate was 300 nL/min over 120 min. Mobile phase A contained 0.1% formic acid in water, while mobile phase B contained 0.1% formic acid in ACN. The LC gradient was as follows: solvent B was maintained at 4% for the first 8 min and was increased from 4 to 10% over 3 min, increased from 10 to 25% over 77 min, increased from 25 to 50% over 10 min, increased from 50 to 99% over 4 min, maintained at 99% for 6 min, decreased from 99% to 4% over 4 min and finally maintained at 4% over 8 min.

Label-free mass spectrometry was performed with a Thermo Orbitrap Fusion (Thermo Fisher Scientific, Beijing, China) mass spectrometer. The scan events were set as follows: full MS scan over a range of 250–1450 *m*/*z* at a mass resolution of 120,000, followed by a CID MS/MS scan repeated on the 20 most intense ions selected from the previous full MS scan with an isolation window. The normalized collision energy was set to 30%, and the activation time was 50 ms. The second stage used the linear ion trap in fast mode for data acquisition with automatic gain control (AGC) of 7000, maximum injection time of 35 ms, and dynamic exclusion time of 18 s. The database used in this experiment was the Uniprot_HUMAN (2019.4.20 Download, https://www.uniprot.org, accessed on 1 April 2021) database. The resulting MS/MS data were processed using MaxQuant 1.5.2.8 (https://www.maxquant.org, accessed on 1 April 2021).

The identification parameters were set as follows: precursor ion mass tolerance, ± 15 ppm; fragment ion mass tolerance, ±0.5 Da; max missed cleavages, 2; static modification, carboxyamidomethylation (57.021 Da) of cysteine residues; and dynamic modifications, oxidative modification (15.995 Da) of methionine residues. To assess the effect of cold stress on the protein profiles of the three blueberry cultivars, we calculated the normalized protein abundance for each of the three blueberry cultivars at the same stage and for the same blueberry cultivar at different stages. According to the *p* value of the primary data, differences with *p* ≤ 0.05 and a difference ratio of ≥1.2 were considered statistically significant and biologically relevant.

### 4.5. Real-Time PCR Analysis

First-strand cDNA was synthesized using the HiScript 1st Strand cDNA Synthesis Kit (Perfect Real Time) (AORT-0060; Gizen organism, Shanghai, China) following the manufacturer’s instructions. qPCR was performed using AceQ qPCR SYBR Green Master Mix (Q121-02; Gizen organism, Shanghai, China) on a LightCycler 480 II Touch fluorescence quantitative PCR instrument (Touch, Beijing, China). The RT-qPCR reaction was performed in a 10 μL system with *VcUBC28* as the internal reference [70]. The primers used are shown in Table S12. The cycle threshold (Ct) 2^−ΔΔCT^ method was used for the relative quantification of mRNA. The reaction was set up for three repetitions.

### 4.6. Statistical Analysis

Statistical analysis was conducted by using Microsoft Excel 2020 (https://www.microsoftstore.com.cn/software/office, accessed on 1 April 2021) and SPSS (SPSS. Inc., Chicago, IL, USA, version 20.0, https://www.ibm.com/cn-zh/spss?lnk=flatitem, accessed on 12 March 2020). Statistical significance was evaluated by a one-way analysis of variance followed by Duncan’s multiple range test. Data are expressed as means ± standard deviations (SDs).

## Figures and Tables

**Table 1:** The numbers of DEGs in the three cultivars during the five developmental stages.

DEG Set	DEG Number	Upregulated	Downregulated
A11_A12_A13_vs_A21_A22_A23	193	50	143
A11_A12_A13_vs_A41_A42_A43	1616	725	891
A11_A12_A13_vs_A51_A52_A53	1566	705	861
A11_A12_A13_vs_A61_A62_A63	1263	587	676
A11_A12_A13_vs_B11_B13_B12	8793	4252	4541
A11_A13_A12_vs_C11_C12_C13	6946	3493	3453
A21_A22_A23_vs_A41_A42_A43	931	485	446
A21_A22_A23_vs_A51_A52_A53	884	453	431
A21_A22_A23_vs_A61_A62_A63	640	351	289
A21_A22_A23_vs_B21_B22_B23	10,082	4802	5280
A21_A22_A23_vs_C21_C22_C23	7300	3601	3699
A41_A42_A43_vs_A51_A52_A53	65	28	37
A41_A42_A43_vs_A61_A62_A63	174	99	75
A41_A42_A43_vs_B41_B42_B43	10,428	4909	5519
A41_A42_A43_vs_C41_C42_C43	7602	3786	3816
A51_A52_A53_vs_A61_A62_A63	78	38	40
A51_A52_A53_vs_B51_B53_B52	10,448	4814	5634
A51_A52_A53_vs_C51_C52_C53	7257	3410	3847
A61_A62_A63_vs_C61_C62_C63	10,676	5409	5267
A62_A61_A63_vs_B61_B62_B63	6339	3506	2833
B11_B12_B13_vs_B21_B22_B23	620	195	425
B11_B12_B13_vs_B41_B42_B43	1307	406	901
B11_B12_B13_vs_B51_B52_B53	2686	826	1860
B11_B12_B13_vs_B61_B62_B63	2579	1399	1180
B11_B12_B13_vs_C11_C12_C13	7610	3850	3760
B21_B22_B23_vs_B41_B42_B43	406	124	282
B21_B22_B23_vs_B51_B52_B53	1132	328	804
B21_B22_B23_vs_B61_B62_B63	2579	1752	827
B22_B23_B21_vs_C21_C22_C23	9710	4953	4757
B41_B42_B43_vs_B51_B52_B53	319	99	220
B41_B42_B43_vs_B61_B62_B63	1305	1094	211
B41_B42_B43_vs_C41_C42_C43	8221	4248	3973
B51_B52_B53_vs_B61_B62_B63	1654	1355	299
B52_B53_B51_vs_C51_C52_C53	9224	4747	4477
B61_B62_B63_vs_C61_C62_C63	8885	4198	4687
C11_C12_C13_vs_C21_C22_C23	282	100	182
C11_C12_C13_vs_C41_C42_C43	928	365	563
C11_C12_C13_vs_C51_C52_C53	3080	1148	1932
C11_C12_C13_vs_C61_C62_C63	5004	2419	2585
C21_C22_C23_vs_C41_C42_C43	637	279	358
C21_C22_C23_vs_C51_C52_C53	2746	1107	1639
C21_C22_C23_vs_C61_C62_C63	5511	2826	2685
C41_C42_C43_vs_C51_C52_C53	740	329	411
C41_C42_C43_vs_C61_C62_C63	5179	2790	2389
C51_C52_C53_vs_C61_C62_C63	5470	3074	2396

Note: A, B, and C represent the ‘Northland’, ‘Bluecrop’, and ‘Berkeley’ cultivars, respectively. The first digit after the letter represents the sampling stage, and the second digit represents biological duplication.

## Data Availability

All the raw and analyzed RNA-seq data applied in this work were released on SRA Run Selector, with the SRA accession number PRJNA910836 (NCBI BioProject, https://www.ncbi.nlm.nih.gov/Traces/study/?acc=PRJNA910836, accessed on 6 April 2023).

## Supplementary Materials

The following supporting information can be downloaded at https://www.mdpi.com/article/10.3390/plants13141911/s1, Figure S1: The ‘Northland’ cultivar suffering from cold damage in the winter and moisture loss in the early spring, causing poor fruit development and falling (A,B). Under field cultivation conditions, the ‘Northland’ cultivar was not protected during overwintering (from December). The photos were taken on 17 May 2019; Figure S2: Principal component analysis of the three blueberry cultivars. Figure S3: Venn diagram of the differentially expressed proteins of the three cultivars at 4 developmental stages including stage 1 (A), stage 2 (B), stage 4 (C), and stage 5 (D); Figure S4: Venn diagram of the differentially expressed proteins for each cultivar. ‘Northland’ (A), ‘Bluecrop’ (B), and ‘Berkeley’ (C). The 4 developmental stages are stage 1, stage 2, stage 4, and stage 5, respectively. Table S1: Weather conditions in Zhuanghe City (2019.12–2020.3); Table S2: Statistical summary of transcriptome information for the three blueberry cultivars; Table S3: Statistical summary of the DEGs among the ‘Northland’ (A), ‘Bluecrop’ (B), and ‘Berkeley’ (C) cultivars in each stage of cold stress; Table S4: Statistical summary of the DEPs among the ‘Northland’ (A), ‘Bluecrop’ (B), and ‘Berkeley’ (C) cultivars in each stage of cold stress; Table S5: Gene family summary in blueberries; Table S6: The differential expression of the NCA gene family in blueberries; Table S7: The differential expression of the WRKY gene family in blueberries; Table S8: The differential expression of the ERF gene family in blueberries; Table S9: Statistical summary of 106 calcium-related DEGs; Table S10: Statistical summary of the 63 glutathione proteins among the ‘Northland’ (A), ‘Bluecrop’ (B), and ‘Berkeley’ (C) cultivars in each stage of cold stress; Table S11: Sample information for each group and including three biological replicates; Table S12: Primers for the qRT-PCR validation of DEGs.

## Abbreviations

DEGs: differentially expressed genes; DEPs: differentially expressed proteins; KEGG: Kyoto Encyclopedia of Genes and Genomes; GO: Gene Ontology; NR: NCBI nonredundant protein sequences; Pfam: protein family; eggNOG: Clusters of Orthologous Groups of proteins; Swiss-Prot: a manually annotated and reviewed protein sequence database; ROS: reactive oxygen species. NGS: next-generation sequencing; CAT: catalase; POD: peroxidase; SOD: superoxide dismutase; CDPKs: calcium-dependent protein kinases; ONT: Oxford Nanopore Technologies; FLNC: full-length, nonchimaeric; BCA: bicinchoninic acid.
