# Supplementary material for: Integrated Transcriptomic and Proteomic Analysis Reveals Molecular Mechanisms of the Cold Stress Response during the Overwintering Period in Blueberries (Vaccinium spp.)
Source: Plants (Basel). 2024 Jul 11;13(14):1911. doi: 10.3390/plants13141911 (PMC11280072; doi:10.3390/plants13141911)
Supplement: Supplementary file 1 [file plants-13-01911-s001.zip › Supplemental Figure S1.pdf]

A B

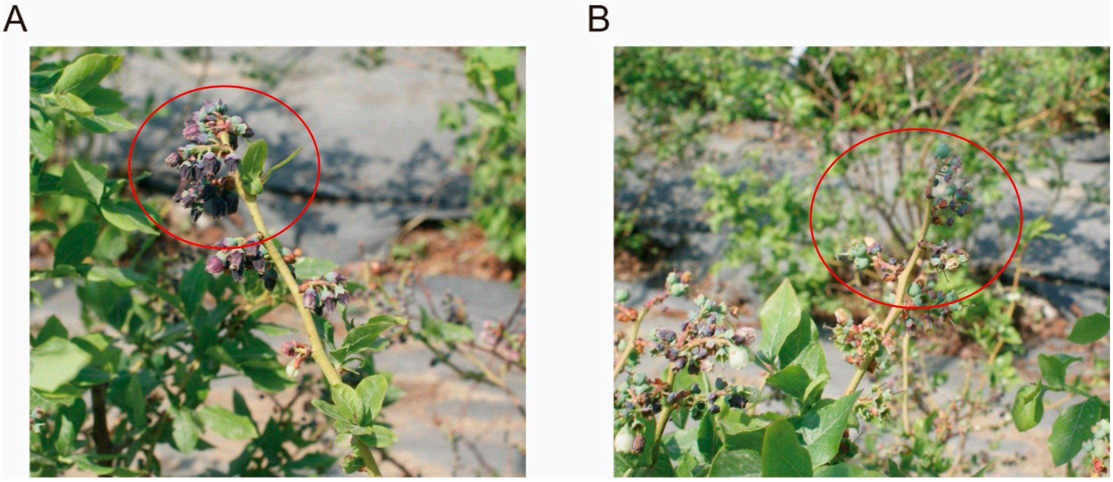

Figure S1 'Northland' cultivar was suffered from cold damage in the winter and lose moisture in the early spring, causing poor fruit development and falling (A-B). Under field cultivation conditions, 'Northland' cultivar was not protected during overwintering (From December). The photos were taken on May 17th, 2019.
