# Supplementary material for: Integrated Transcriptomic and Proteomic Analysis Reveals Molecular Mechanisms of the Cold Stress Response during the Overwintering Period in Blueberries (Vaccinium spp.)
Source: Plants (Basel). 2024 Jul 11;13(14):1911. doi: 10.3390/plants13141911 (PMC11280072; doi:10.3390/plants13141911)
Supplement: Supplementary file 1 [file plants-13-01911-s001.zip › Supplemental Figure S3.pdf]

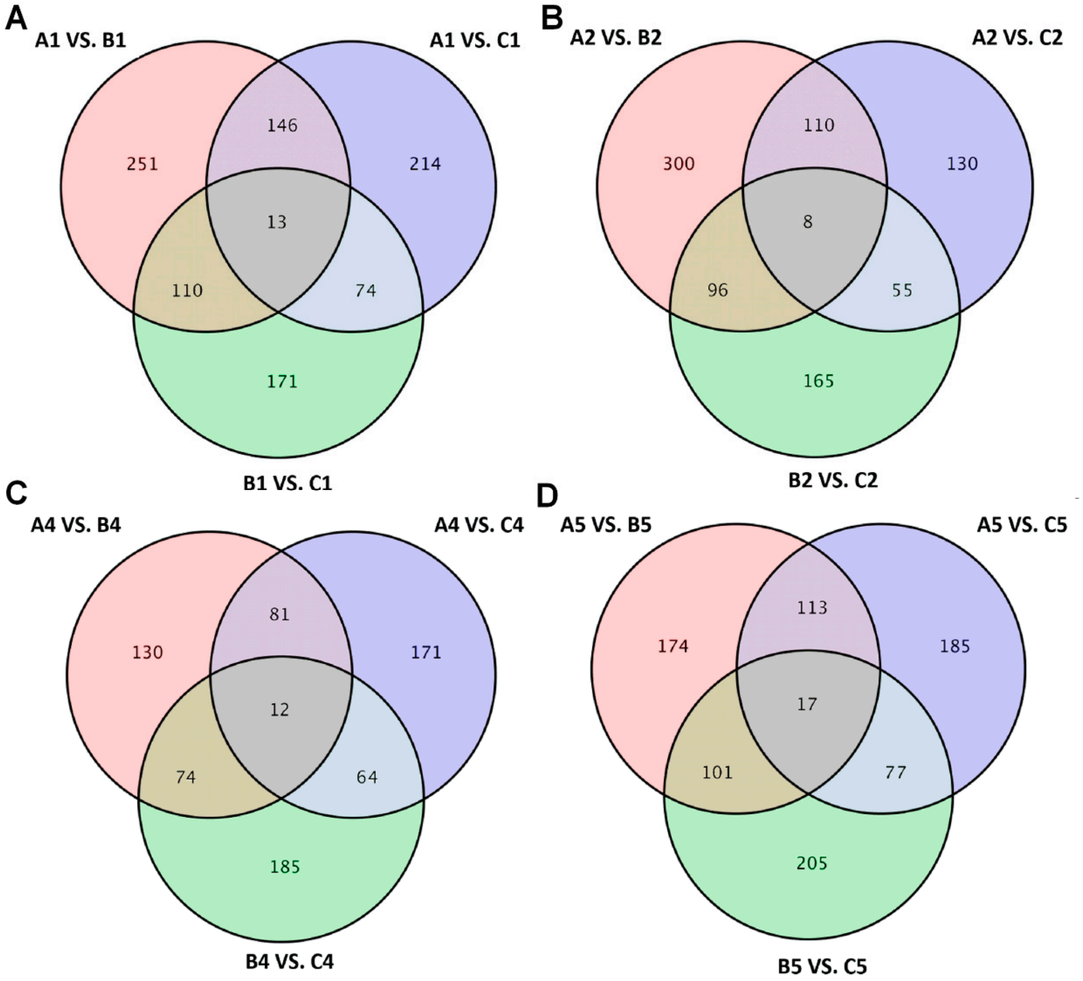

Figure. S3 Venn diagram of the differentially expressed proteins of the three cultivars at the 4 developmental stages. stage 1 (A), stage 2 (B), stage 4 (C) and stage 5 (D).
