# Supplementary material for: Integrated Transcriptomic and Proteomic Analysis Reveals Molecular Mechanisms of the Cold Stress Response during the Overwintering Period in Blueberries (Vaccinium spp.)
Source: Plants (Basel). 2024 Jul 11;13(14):1911. doi: 10.3390/plants13141911 (PMC11280072; doi:10.3390/plants13141911)
Supplement: Supplementary file 1 [file plants-13-01911-s001.zip › Supplemental Figure S4.pdf]

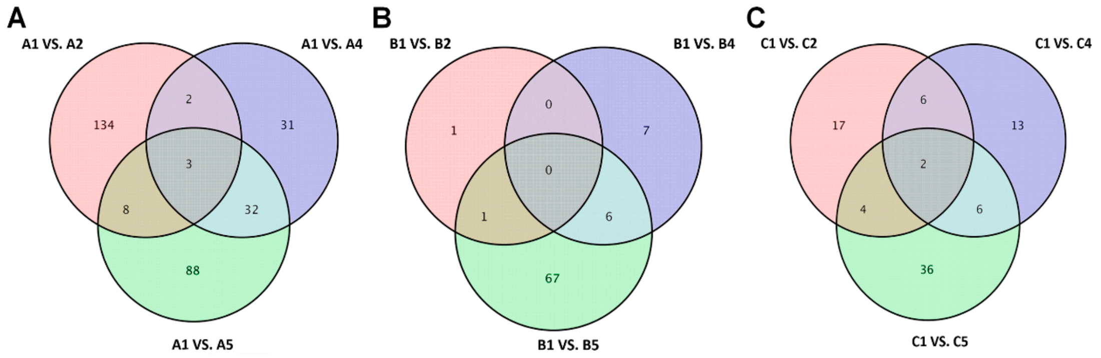

Figure S4 Venn diagram of the differentially expressed proteins for each cultivar. 'Northland' (A), 'Bluecrop' (B) and 'Berkeley' (C). The 4 developmental stages were stage 1, stage 2, stage 4 and stage 5, respectively.
