# Supplementary material for: Integrated Transcriptomic and Proteomic Analysis Reveals Molecular Mechanisms of the Cold Stress Response during the Overwintering Period in Blueberries (Vaccinium spp.)
Source: Plants (Basel). 2024 Jul 11;13(14):1911. doi: 10.3390/plants13141911 (PMC11280072; doi:10.3390/plants13141911)
Supplement: Supplementary file 1 [file plants-13-01911-s001.zip › Supplementary Table S11.pdf]

Table S11 Sample information for each group and including three biological replicates

Cultivars	Sampling time	Stage	Group name	Transcriptome	Proteome
'Northland'	2019,12,14	1	A1	A1	A1
	2019,12,28	2	A2	A2	A2
	2020,01,05	3			
	2020,01,17	4	A4	A4	A4
	2020,02,10	5	A5	A5	A5
	2020,03,05	6	A6	A6	
	2020,03,20	7			
'Bluecrop'	2019,12,14	1	B1	B1	B1
	2019,12,28	2	B2	B2	B2
	2020,01,05	3			
	2020,01,17	4	B4	B4	B4
	2020,02,10	5	B5	B5	B5
	2020,03,05	6	B6	B6	
	2020,03,20	7			
'Berkeley'	2019,12,14	1	C1	C1	C1
	2019,12,28	2	C2	C2	C2
	2020,01,05	3			
	2020,01,17	4	C4	C4	C4
	2020,02,10	5	C5	C5	C5
	2020,03,05	6	C6	C6	
	2020,03,20	7			
