# Supplementary material for: Integrated Transcriptomic and Proteomic Analysis Reveals Molecular Mechanisms of the Cold Stress Response during the Overwintering Period in Blueberries (Vaccinium spp.)
Source: Plants (Basel). 2024 Jul 11;13(14):1911. doi: 10.3390/plants13141911 (PMC11280072; doi:10.3390/plants13141911)
Supplement: Supplementary file 1 [file plants-13-01911-s001.zip › Supplementary Table S12.pdf]

**Table S12** Primers for qRT-PCR validation of DEGs

| Gene name | Primer Sequence （5'-3'） | |
|---|---|---|
| | Forward | Reverse |
| *maker-VaccDscaff22-snap-gene-28.43* | AGATGTGAATTGCGCCAGGA | CTTGATGGATTCGCCTGGGT |
| *maker-VaccDscaff28-augustus-gene-41.19* | CTAGGATACAGGACGAAAGCAAA | GCAAGTCACAACCGTAAAGAACT |
| *maker-VaccDscaff33-augustus-gene-305.26* | CAACCACTCCAGCCAACTGA | CAAGGCTGAGCAAGGTCACT |
| *maker-VaccDscaff42-augustus-gene-108.22* | CAATGCCAACCCAACGAGTG | GGGGAAAGGTCTTCTGGTCG |
| *maker-VaccDscaff48-augustus-gene-37.45* | CTGATGAACCCTCGTCCTCG | CGACGGTGTCATCTCTCCTG |
| *maker-VaccDscaff49-snap-gene-45.56* | AGGGAGTTCATTTGGGGCAG | GCACAAGGTTGGACGATTGG |
| *maker-VaccDscaff51-augustus-gene-12.23* | ACCAAACCCAAGCCTTCCAA | GGTACACTCGTGGGAGCTTT |
| *maker-VaccDscaff61-augustus-gene-0.32* | CCACAAGCACTACGCATCAC | TGAAGCAAAGGTCTGGGTGAA |
| *maker-VaccDscaff9-snap-gene-31.79* | CAACCATAAACGATGCCGA | AGCCTTGCGACCATACTCC |
