# Supplementary figures and images for: Integrated Transcriptomic and Proteomic Analysis Reveals Molecular Mechanisms of the Cold Stress Response during the Overwintering Period in Blueberries (Vaccinium spp.)
Source: Plants (Basel). 2024 Jul 11;13(14):1911. doi: 10.3390/plants13141911 (PMC11280072; doi:10.3390/plants13141911)

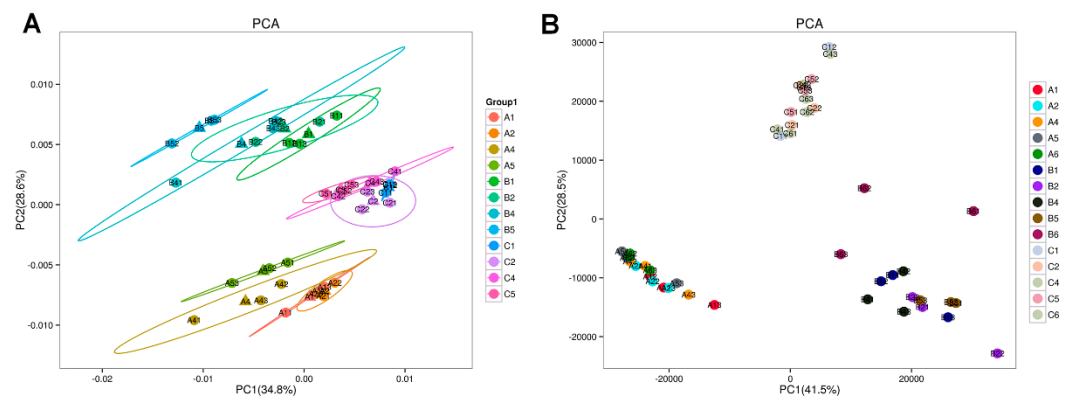

**Figure S2** Principal component analysis of the three blueberry cultivars.

Supplement: Supplementary file 1 [file plants-13-01911-s001.zip › Supplemental Figure S2.pdf]
